# Different Spatial Characteristic Changes in Lumbopelvic Kinematics Before and After Fatigue: Comparison Between People with and Without Low Back Pain

**DOI:** 10.3390/bioengineering12030214

**Published:** 2025-02-20

**Authors:** Xin Xi, Ling Zhang, Haixin Yu, Yifei Qin, Long Jia, Tsung-Yuan Tsai, Yan Yu, Liming Cheng

**Affiliations:** 1 Department of Spine Surgery, Tongji Hospital, School of Medicine, Tongji University, Shanghai 200092, China; 1931191@tongji.edu.cn (X.X.); 1950329@tongji.edu.cn (Y.Q.); 2 Key Laboratory of Spine and Spinal Cord Injury Repair and Regeneration, Ministry of Education, Department of Spine Surgery, Tongji Hospital, School of Medicine, Tongji University, Shanghai 200092, China; 3 School of Exercise and Health, Shanghai University of Sport, 200th. Hengren Road, Yangpu District, Shanghai 200438, China; yiyangqx2000@163.com; 4 Department of Orthopedic Surgery, Yangpu Hospital, School of Medicine, Tongji University, Shanghai 200090, China; 2133192@tongji.edu.cn; 5 Department of Orthopedics, QingPu Branch of Zhongshan Hospital Affiliated to Fudan University, Shanghai 201799, China; jialong19880523@163.com; 6 School of Biomedical Engineering, Shanghai Jiao Tong University, Shanghai 200030, China; tytsai@sjtu.edu.cn; 7 TaoImage Medical Technologies Corporation, Shanghai 201204, China

**Keywords:** low back pain, lumbopelvic kinematics, fatigue, forward-backward bending, dual fluoroscopy

## Abstract

Background: The lumbopelvic region plays a pivotal role in enabling various functional activities. This study quantified and compared the kinematic changes between healthy individuals and patients with recurrent low back pain (LBP) in both rested and fatigued states to gain insight into the kinematic adaptation and mechanisms underlying kinematic variations that occur in the presence of these factors. Methods: Participants were divided into two groups: the LBP (*n* = 23) and healthy control groups (*n* = 19). Dynamic lumbopelvic measurements were taken using a biplane radiography image system while the participants performed weight-bearing forward-backward bending before and after fatigue. All lumbopelvic kinematics were described as the three-dimensional motion of the vertebra relative to the pelvis and were measured at normalized time intervals from maximum extension to approximately 45° of flexion. Results: Repetitive lifting- and lowering-induced fatigue significantly affected lumbopelvic kinematics in the anterior/posterior translation (mm) and rotation around the z-axis in both healthy individuals and patients with LBP (*p* < 0.05). In healthy individuals, significant differences occurred in approximately 13–83% of the forward-backward bending cycle (0–100%), whereas, in patients with LBP, significant differences mainly occurred in 61–93% of the cycle (*p* < 0.01). Conclusions: The lumbopelvic kinematic changes observed in both LBP patients and healthy individuals after fatigue may indicate protective compensation or vulnerability and could play a role in LBP dysfunction.

## 1. Introduction

Low back pain (LBP) is a prevalent, disabling global health concern that significantly affects individuals’ quality of life and productivity, with substantial societal and economic impacts [1,2,3]. In the case of certain individuals with LBP, abnormal motor patterns and postural behaviors may constitute the mechanical foundation underlying their pain symptoms [4]. These changes can influence the motor functioning of the spine and other joints, exacerbate existing pain, and contribute to the development of further disability [5,6,7]. Thus, obtaining a thorough understanding of the kinematic characteristics and underlying mechanistic variations of LBP is a crucial and urgent task, one that is essential for facilitating the development of more effective intervention and management strategies.

The significance of assessing spinal kinematics in advancing our understanding of the mechanical factors implicated in LBP is universally acknowledged [8,9,10]. Lifting has been established as a significant risk factor for the onset of LBP [11], yet it remains an essential activity for maintaining individual independence for many [12]. Changes in spinal movements while lifting are anticipated in individuals with LBP; however, these kinematic alterations are inconsistent and pose clinical assessment challenges. The precise impact of a prior history of LBP on lifting, particularly in terms of potential adaptive neuromuscular alterations versus concurrent pain, remains uncertain. Moreover, while it is intuitive to focus on lumbar spine movements as the primary site of dysfunction, research has demonstrated a close synchronization between the lumbar spine and the hip/pelvis [13], as well as between the lumbar and lower thoracic regions [14], during functional activities. Neglecting adjacent areas to a symptomatic spinal segment may result in missing crucial information regarding their functional behaviors [15].

The local dynamic kinematics of the lumbopelvic region are significantly influenced by various factors, including fatigue and LBP [15,16,17]. Specifically, the interplay between fatigue and LBP can be particularly complex because both factors can independently and synergistically influence the local dynamic kinematics of the lumbopelvic region [18,19,20]. Previous studies have investigated the effects of fatigue and LBP on various aspects of neuromuscular control and postural stability [15,19,20,21,22,23,24]. When experienced cumulatively, fatigue can lead to decreased muscular endurance and increased muscle fatigability, resulting in altered joint kinematics and postural stability [25,26]. Particularly, the lumbopelvic region is highly susceptible to these changes as it bears significant loads during functional activities such as lifting, bending, and twisting [27,28]. Notably, increasing evidence suggests that fatigue within the sensorimotor system drives kinematic changes in lifting strategies [20,29]. Reduced sensorimotor feedback during cyclic lifting may decrease spinal stability [20]. This chain of events may facilitate the fatigue-related biomechanical changes observed in lifting strategies [20]. Furthermore, local dynamic kinematics of the lumbopelvic region before and after functional activities, which involve complex movement patterns and rapid changes in loading conditions, remain poorly understood. Understanding the characteristics of these regional kinematics, particularly under different conditions, such as before and after fatigue, is crucial for developing effective treatment strategies and preventing further deterioration of lumbopelvic function.

Recent research has revealed that patients with LBP tend to adopt stiff strategies to control the lumbar-pelvic region during functional movements such as walking and flexion/extension (F/E) [30,31]. This may lead to difficulties in isolating adjacent segmental movements or delays in lumbar-pelvic motion during multi-segmental tasks. However, evidence suggests that premature and excessive lumbar-pelvic motion occurs during lower limb movements [32,33]. Based on theories of musculoskeletal injury, excessive lumbar-pelvic motion during lower limb activities can lead to the accumulation of soft tissue stress, resulting in microtrauma and ultimately eliciting LBP symptoms [34]. Nevertheless, focusing solely on the increase and advancement of lumbar-pelvic motion while neglecting changes such as motion delays and decreased stability may overlook these manifestations as potential indicators of stiff strategies protecting painful structures. Researchers hypothesize that repeated early lumbar-pelvic motion in the same direction during daily activities may eventually lead to excessive motion [35]. However, discussions on the factors associated with different lumbar-pelvic motion patterns remain inadequate. Therefore, these conflicting theories require further in-depth investigation to clarify their intrinsic relationships and differences.

To advance the variability-fatigue and stiff strategies hypotheses, this study aimed to assess and compare different kinematic characteristic changes in the lumbopelvic region before and after fatigue between individuals with and without LBP. By quantifying and comparing the lumbopelvic kinematic changes between healthy individuals and patients with LBP, both in rested and fatigued states, we aimed to gain insights into the kinematic adaptation that occurs in the presence of these factors. We hypothesized that fatigue would significantly affect the kinematic variables and decrease stability within the lumbopelvic region, while individuals with LBP would demonstrate distinct kinematic deviations compared to healthy controls. Using an advanced dynamic biplane radiographic imaging system and biomechanical analysis, we quantified these kinematic changes and explored the underlying biomechanical mechanisms responsible for these kinematic changes and disparities.

## 2. Materials and Methods

### 2.1. Study Participants

This research was undertaken with prior authorization from the Institutional Review Board (IRB) of Tongji Hospital in Shanghai, China (Protocol No. 2021-011-SK), and was conducted in strict adherence to the ethical guidelines stipulated in the Helsinki Declaration [36]. Recruitment procedures were executed within the personal networks and workplace settings of the investigating researchers. Prior to the collection of any personal or health-related data, all participants were required to provide written informed consent.

The inclusion criteria were as follows: aged between 18 and 40 years, ability to perform the required lifting and forward-backward bending tasks, and sufficient understanding of the Chinese language. Participants were excluded if they had (1) spinal deformity; (2) pain extending to and/or beyond the gluteal fold; (3) previous spine, hip, or knee surgery; (4) spinal root pain; (5) spinal tumors or infections; (6) neurological symptoms such as weakness, tingling, or anesthesia in the lower extremities; and (7) visual or hearing impairment. Participants were referred by an orthopedic specialist, and an experienced physical therapist assessed whether they met the exclusion criteria. In order to better analyze and compare the results, we also recruited a corresponding number of asymptomatic subjects (aged 18–40 years) as a healthy control (HC) group.

**Figure 1:**
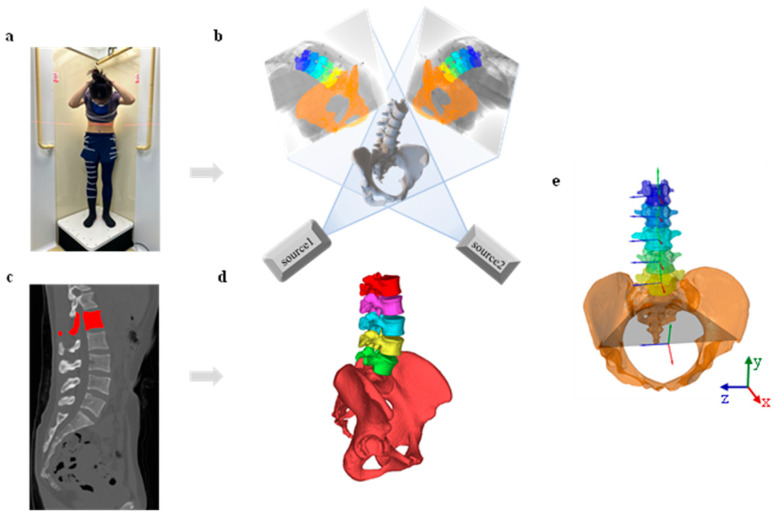
Biplane radiography data collection and processing. (**a**) Participants performed full range of motion (ROM) extension to approximately 45°of flexion while synchronized biplane radiographs were collected (image resolution: 2804 × 2804 pixels). (**b**) Accurate lumbar-pelvic positions were reconstructed using a virtual dual fluoroscopic system. (**c**) CT scans from L1 to the pelvis were collected and (**d**) used to create 3D bone models. (**e**) The axes of rotation for the joint coordinate system of the lumbar and pelvic segments were established to calculate six degrees of freedom (6-DOF) kinematics throughout the full ROM.

All participants underwent a lumbopelvic computed tomography (CT) scan (SOMATOM Definition AS1; Siemens, Shanghai United Imaging Healthcare Co., Ltd, uCT760, 120 kV and 80 mA) in a supine, relaxed position (thickness, 0.6 mm; resolution, 512 × 512 pixels, pixel spacing 0.8 × 0.8 mm). The CT images were imported and segmented into three-dimensional (3D) bone models of the lumbar spines and pelvis in the 3D modeling software Amira 6.7 (Thermo Fisher Scientific, Rockford, IL, USA) (Figure 1c,d).

### 2.2. Experiment Protocol

**Figure 2:**
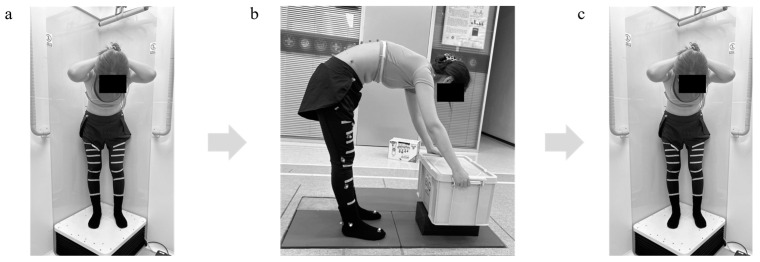
Overview of the experimental steps. (**a**) Step one, dynamic kinematics monitoring of the lumbopelvic before fatigue, (**b**) Step two, repetitive lifting and lowering-induced fatigue (Borg ≥ 17), (**c**)Step three, dynamic kinematics monitoring of the lumbopelvic after fatigue.

The experiment was conducted in three sessions (Figure 2). In the first session, each participant performed weight-bearing forward-backward bending from maximum extension to approximately 45° of flexion to keep the participants within the view of the dynamic biplane radiography image system (TaoImage, Shanghai, China; image resolution 2804 × 2804 pixels). During the forward-backward bending, participants were instructed to position their palms in front of their lower extremities and proceed in an orderly fashion along a designated marking line. They crossed their hands behind their heads and held them still, and biplanar images of the lumbar vertebrae at different bending angles were recorded synchronously (Figure 1a). The participants kept their feet shoulder-width apart and were trained to maintain both knees straight throughout the process. To familiarize the participants with the tasks, they practiced the activities before the actual testing. The test was repeated if participants deviated from the task instructions, resulting in invalid trials. Throughout the experiment, all participants were required to wear lead-protective clothing in non-collection areas to minimize unnecessary radiation exposure.

After completing the first session, the participants were placed on the motion capture systems to perform repetitive lifting and lowering while standing at the center of a force platform (AMTI, Watertown, MA, USA) until they reported a score of 17 on the Borg scale, indicating the highest safe level of fatigue and task-stopping point [15]. A score of 17 corresponded to “very difficult” on the Borg scale. Considering this highly demanding task, a score of 17 was established as the wrap-up point for task repetition to protect the participants, particularly patients, from the risk of possible injuries. All participants were taught how to use the Borg scale with a target of 17 (“very difficult”) before data acquisition. Subsequently, they performed several lifting-lowering movements to familiarize themselves with the testing session. Real-time visual monitoring and post-session statistical assessment were used to eliminate significant changes in technique and timing.

All participants were given the following verbal instructions: “Perform a box lift as you would in your daily life, holding both handles located on the sides of the box naturally”. Each lift was commenced in the standing position. The participants leaned forward to reach and grasp the box, and they fully extended their knees and hips to return to a standing position for 2 s while carrying the box (weight: 5 kg) [37]. All lifting trials started with the feet parallel about hip width apart and 15 cm behind the box. The box had to be grabbed with both hands, lifted up with the elbows extended or slightly flexed (height of the handles in upright standing position: about hip/pelvis height), and placed back on the same place [38].They were also instructed to keep their heels on the ground and maintain a broadly consistent lifting style throughout the task. Lifting was performed at a self-determined pace, with the natural lifting frequency established during practice and subsequently maintained using an auditory metronome. No physical constraints were placed on the pelvis and arms to mimic the functional and natural spine.

After completing the above functional activities to achieve the task-stopping point of exercise-induced fatigue, the participants performed the last session, returned to the dynamic biplane radiography image system, and repeated all the movements from the first part.

### 2.3. In Vivo Kinematics Measurements

The anatomical coordinate systems of the lumbar spine and pelvis were defined according to the guidelines of the International Society of Biomechanics [39].

(1) The origin of the coordinate system at each vertebral level was placed at the midpoint between the upper and lower endplates. The proximal/distal (PD) axis was aligned with the line connecting the centers of these endplates. The medial/lateral (ML) axis was defined as a line parallel to the one joining the right and left pedicles, pointing to the right. The anterior/posterior (AP) axis was perpendicular to both the PD and ML axes and pointed anteriorly [40].
(2) For the pelvic segment, the origin of the coordinate system was located midway between the left and right anterior superior iliac spines (ASISs), and the ML axis was the line connecting the right and left ASISs, pointing to the right. The AP axis was defined as the line parallel to a line lying in the plane defined by the two ASISs and the midpoint of the two posterior superior iliac spines, orthogonal to the z-axis and pointing anteriorly. The PD axis was perpendicular to both AP and ML axes, pointing cranially [41] (Figure 1e).

### 2.4. Data Processing and Analysis

Owing to the limitations of the paper length, it is not feasible to present all the data analysis and processing procedures within a single document. Therefore, this study solely describes the lumbar-pelvic kinematics results and analytical methods for the two groups of participants before and after exercise-induced fatigue.

Pairs of radiographic images of the lumbar spine and pelvis were analyzed from maximum extension to 45° flexion to evaluate the kinematics of the lumbar region relative to the pelvis in 6DOF. The 3D lumbar and pelvic models and two-dimensional (2D) fluoroscopic images were imported into MATLAB (R2018a; MathWorks, Natick, MA, USA) for a 2D to 3D registration procedure, as previously reported [40]. The 3D lumbar and pelvic models were independently manipulated in 6DOF until their projection best matched the outlines of the 2D paired fluoroscopic images (Figure 1b). The current positions of the models were considered to reproduce the in vivo 3D positions of the lumbopelvic joint, and the lumbopelvic kinematics during the actual motion were obtained with a 1 mm precision in translation and 1° in rotation [42]. All lumbar kinematics were described as the 3D motion of the lumbar vertebra relative to the pelvis and measured at normalized time intervals from maximum extension to approximately 45° of flexion.

### 2.5. Statistical Analysis

The normality of the kinematics was assessed using the Shapiro–Wilk test. Statistical parametric mapping (SPM) was used to investigate the differences in kinematic changes before and after exercise-induced fatigue at different normalized times. One-dimensional SPM (SPM1D) was performed in MATLAB (MATLAB, 2022b; MathWorks, Natick, MA, USA) using an open-access code from spm1d.org. For non-normal data, statistical non-parametric mapping was performed. Continuous variables are presented as the mean and standard error. Statistical significance was set at an alpha level of 0.05 for all comparisons.

## 3. Results

A total of 19 pain-free adults (males, 10; females, 9; age, 22.53 ± 2.41 years; body mass index [BMI], 19.97 ± 1.67 kg/m^2^) and 23 patients with LBP (male, 11; female, 12; age, 24.10 ± 2.12 years; BMI, 21.51 ± 2.15 kg/m^2^) were included in this study. All participants completed the experimental task. The participant characteristics and clinical features are shown in Table 1. No significant differences were observed between the LBP and HC groups in terms of height or weight.

### 3.1. Local Dynamic Kinematic Changes with Fatigue

The SPM results indicated that repetitive lifting- and lowering-induced fatigue had a significant effect on lumbopelvic kinematics in the AP translation (mm) and rotation around the z-axis in both healthy individuals and patients with LBP (*p* < 0.05). As expected, from maximum extension to approximately 45° flexion, all participants experienced reduced AP displacement and rotation around the z-axis after fatigue (*p* < 0.01). Moreover, before and after fatigue, the lumbopelvic kinematics of patients with LBP were lower than those of healthy individuals. Specifically, in healthy individuals, the average anterior translation of L1 to the pelvis before and after fatigue was −90.58 ± 21.76 mm vs. −83.86 ± 24.73 mm (*p* < 0.01). Conversely, for patients with LBP, the average anterior translation of L1 to the pelvis before and after fatigue was −88.12 ± 21.04 mm vs. −82.10 ± 22.61 mm (*p* < 0.01). In terms of rotation angle changes, the average flexion of L1 to the pelvis in healthy individuals and patients with LBP before and after fatigue were −4.7 ± 7.9° vs. −0.9 ± 8.7° (*p* < 0.01) and −3.3 ± 8.4° vs. 0.4 ± 9.8° (*p* < 0.01), respectively. As shown in Table 2, the average displacement of L1 to L4 relative to the pelvis in the AP direction showed a decreasing trend before and after fatigue, both in healthy individuals and patients with LBP. However, the average displacement of L5 relative to the pelvis in the AP direction showed a small increase compared with that of L4. After fatigue, the values in the rotation of F/E decreased at L1–L4 relative to the pelvis but increased at L5 relative to the pelvis before fatigue. However, no apparent trend of increase or decrease was observed in rotation around the z-axis after fatigue for both healthy individuals and patients with LBP. Kinematic changes in other DOF of the lumbopelvic are provided in Supplementary Materials.

### 3.2. Comparison of the Period of a Significant Difference in Lumbopelvic Kinematics Between the LBP and HC Groups

**Figure 3:**
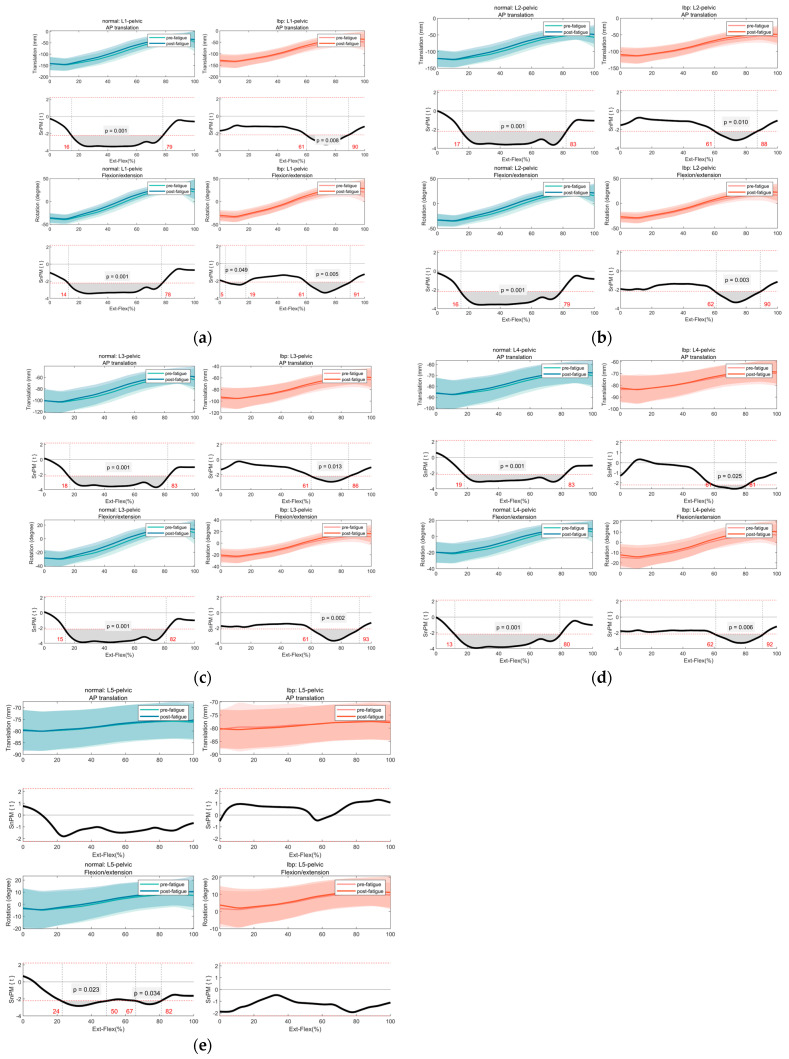
(**a**−**e**) Comparison of lumbopelvic kinematics (AP translation and flexion/extension (F/E) angular rotation) in conditions before and after fatigue between the LBP and HC groups.

Figure 3a–e shows a comparison of lumbopelvic kinematics before and after fatigue between the LBP and HC groups. As previously stated, both healthy individuals and patients with LBP showed significant differences in lumbopelvic kinematics before and after fatigue. However, in healthy individuals, significant differences occurred in approximately 13–83% of the forward-backward bending cycle (0–100%). Conversely, in patients with LBP, significant differences mainly occurred in 61–93% of the cycle (*p* < 0.01). Notably, the aforementioned disparities were exclusively identified in the kinematics of the lumbar vertebrae L1–L4 relative to the pelvis. No significant differences were observed, before or after fatigue, in the AP displacement or F/E angular rotation of L5 to the pelvis between healthy participants and those with LBP.

## 4. Discussion

This study aimed to investigate the local dynamic kinematics of the lumbopelvic complex before and after functional activities, focusing on the effects of fatigue and LBP. While the use of dual fluoroscopy provided precise imaging results, it is important to acknowledge the limitations of these findings when interpreting functional changes associated with spine overload. Imaging techniques, such as dual fluoroscopy, are valuable tools for quantifying lumbar-pelvic kinematics. However, they can only provide one aspect of the complex biomechanical changes occurring during functional activities. Our findings should be considered as indicators of potential functional adaptations rather than definitive evidence of functional changes.

Despite these limitations, our study revealed notable discrepancies in kinematic characteristics between individuals with LBP and healthy controls (HCs) across various bending angles and ranges of flexion before and after fatigue. Specifically, in healthy individuals, significant differences occurred in approximately 13–83% of the forward-backward bending cycle (0–100%); conversely, in patients with LBP, these mainly occurred in 61–93% of the cycle (*p* < 0.01). Therefore, healthy individuals demonstrated the ability to make adaptive kinematic adjustments at smaller angles of flexion and across a broader range of flexion compared to those with LBP. These findings align with those of previous research, concluding that fatigue alters lumbar-pelvic kinematics, and individuals without pain are better able to adapt to fatigue, maintaining more complex movement patterns [15]. This may suggest that individuals with LBP do not select movement strategies that consider the varying demands of functional tasks. Their rigid lumbar-pelvic movement patterns (or stiff strategies) may represent an adaptive strategy to avoid exacerbating their pain. However, this could also constitute a maladaptive mechanism, as it may contribute to the chronicity and recurrence of LBP [35].

Our findings indicated that, in comparison to healthy participants, patients with LBP exhibit fewer kinematic changes in the lumbo-pelvic region before and after fatigue. This aligns with previous research suggesting that post-fatigue, LBP patients may adopt a more synchronized lumbo-pelvic movement pattern (a more coordinated movement between the lumbar spine and pelvis) to compensate for reduced lumbar spine stability [43,44,45,46]. Notably, the relationship between abnormal lumbo-pelvic kinematics and LBP is bidirectional. Lumbar pelvic kinematic abnormalities can exacerbate existing conditions, such as herniated discs, spinal stenosis, and degenerative disc disease. Additionally, these conditions can further compromise spinal stability and increase the sensitivity of pain receptors in the affected areas, thereby amplifying pain perception [47,48].

Another noteworthy observation is the alteration in kinematic changes at L5 relative to the pelvis in both individuals with LBP and HC before and after fatigue. That is, following fatigue, the kinematic variations of L5 relative to the pelvis do not decrease but rather increase, in contrast to the kinematic changes of L1–L4 relative to the pelvis, with a more pronounced effect observed in the LBP group. This may be attributed to the anatomical characteristics of the lumbar-pelvic region. The facet joints of L1 to L4 in the lumbar spine are almost vertical with sagittal displacement, and those of the L5–S1 segment are closer to the coronal plane, preventing excessive lumbar anterior movement. The kinematic changes in the LBP group at the L5 to pelvis level compared to the L1–L4 to pelvis level may represent a compensation or anatomical adaptation to the decreased motion at the upper lumbar levels.

In normal static and dynamic postures, several trunk muscles and passive structures interact to control and protect the spinal cord. The paraspinal muscles (erector spinae, multifidus, and psoas major) support the spine and act as dynamic stabilizers [49,50]. In patients with LBP in a state of fatigue, these muscles may be unable to provide sufficient stabilization, transferring load sharing to passive structures earlier in trunk flexion [51]. The multifidus (MF) muscle is key in controlling the intervertebral motion of each vertebra in the sagittal plane during flexion and extension of the lumbar spine [52]; specifically, it produces the “rocking” action of each vertebra evident during lumbar spine extension and controls this component during lumbar flexion [53]. However, the MF muscles are not the primary producers of lumbar spine extension torque. In fact, they contribute approximately 20% of the extensor torque at the L4 and L5 vertebral levels. The lumbar erector spinae and thoracic components of the erector spinae contribute 30% and 50%, respectively [54]. Fatigue of the lower back extensor muscles may occur during static flexion owing to prolonged passive stretching of the muscles [55]. When the contribution of creep-deformed passive tissues is reduced, the lower back extensor muscles are required to generate more active forces during weight holding or lifting after static flexion. Prolonged trunk flexion leads to passive tissue deformation, potentially increasing the contribution of trunk muscles to spinal stabilization mechanisms during fatigue [55,56,57], which may be attributed to changes in the relative contribution mechanisms of key paraspinal muscles and passive tissues to spinal stability in patients with LBP compared to healthy individuals. This ultimately leads to delayed adjustment and adaptability to fatigue in these patients.

The interplay between fatigue and LBP in modulating lumbopelvic kinematics is complex and multifaceted [18]. On the one hand, fatigue can compromise the stability of the lumbopelvic region, making it more susceptible to injury and pain [58]; on the other, LBP can lead to altered movement patterns and increased muscle activation, further exacerbating fatigue and pain [18]. This vicious cycle highlights the importance of addressing these factors in the management of LBP. Our results provide insight into the mechanism of kinematic stability of the lumbopelvic joint associated with lifting-induced fatigue and its interaction with LBP. However, it is important to note that the mechanisms underlying the observed changes in lumbopelvic kinematics remain largely unknown. Future studies should investigate the neural and biomechanical factors contributing to these changes, focusing on understanding the roles of pain modulation, muscle co-contraction, and spinal reflexes. Moreover, our results have implications for rehabilitation protocols aimed at improving lumbopelvic stability and reducing the risk of LBP. Strengthening exercises targeting the paraspinal muscles and moderate physical exercise may help improve lumbopelvic control and stability, potentially reducing the risk of injury and pain.

The main limitation of our study is the lack of information on whole-body motion analysis obtained from electromyography and motion capture. Although this limitation is partly offset by the adoption of sensitive, quantitative measures of motion, caution is needed when interpreting the results. Secondly, the evaluation of fatigue in this study was based on the Borg scale, which is a subjective evaluation tool. While this method has been widely used in similar studies, it may not provide an accurate measure of fatigue in all individuals. Therefore, future research should consider using more objective measures of fatigue, such as electromyography or biomechanical analysis, to better understand the impact of fatigue on lumbopelvic kinematics. Finally, to improve the accuracy of the kinematics calculation and reduce unnecessary radiation, we focused on the flexion-extension direction for acquisition and analysis; therefore, the distribution characteristics and quantitative results of lumbopelvic kinematics in other degrees of freedom were lacking. However, assessing the displacement of the lumbar to the pelvis in the full region of the lumbar forward-backward bending could provide a useful reference for estimating 6DOF lumbopelvic motion.

## 5. Conclusions and Future Perspectives

In conclusion, while imaging techniques such as dual fluoroscopy provide valuable information about lumbar-pelvic kinematics, they should be interpreted with caution when drawing functional conclusions. Our study suggests that lumbopelvic kinematic changes observed after fatigue may indicate protective compensation or vulnerability and could play a role in LBP dysfunction. However, more research is needed to fully understand the underlying mechanisms and to develop effective interventions to prevent and treat LBP.

## Figures and Tables

**Table 1:** Participant characteristics and clinical features.

	Mean ± SD Healthy (*n* = 19)	Mean ± SD LBP (*n* = 23)	t	*p* Value
Sex (F/M)	9/10	12/11	-	-
Age (years)	22.53 ± 2.41	24.10 ± 2.12	−2.056	0.046
Height (m)	1.71 ± 0.06	1.72 ± 0.08	−0.252	0.802
Mass (kg)	58.00 ± 7.32	61.20 ± 12.32	−1.962	0.057
BMI (kg/m^2^)	19.97 ± 1.67	21.51 ± 2.15	−2.552	0.015
VAS		4.61 ± 1.95	-	-
ODI		13.13 ± 11.52	-	-

F, female; M, male; BMI, body mass index; VAS, visual analogue scale; ODI, Oswestry Disability Index.

**Table 2:** Statistical results of the lumbopelvic kinematics before and after fatigue between people with and without LBP.

Lumbopelvic Kinematics		Healthy		LBP	
		Pre-Fatigue (Mean ± SD)	Post-Fatigue (Mean ± SD)	Pre-Fatigue (Mean ± SD)	Post-Fatigue (Mean ± SD)
L1 to pelvis	AP (mm)	−90.58 ± 21.76	−83.86 ± 24.73 **	−88.12 ± 21.04	−82.10 ± 22.61 **
	F/E(deg)	−4.68 ± 7.89	−0.86 ± 8.74 **	−3.26 ± 8.44	0.41 ± 9.80 **
L2 to pelvis	AP (mm)	−86.74 ± 19.10	−81.60 ± 20.60 **	−83.30 ± 17.31	−79.40 ± 18.33 **
	F/E(deg)	−6.15 ± 9.23	−2.70 ± 9.39 **	−5.20 ± 7.46	−2.10 ± 8.84 **
L3 to pelvis	AP (mm)	−81.55 ± 15.09	−78.41 ± 15.95 **	−78.75 ± 13.63	−76.55 ± 13.88 *
	F/E(deg)	−8.18 ± 8.25	−5.20 ± 8.55 **	−5.38 ± 7.95	−2.60 ± 9.34 **
L4 to pelvis	AP (mm)	−77.86 ± 11.17	−76.52 ± 11.25 **	−76.45 ± 9.84	−75.74 ± 9.49
	F/E(deg)	−6.38 ± 9.57	−4.25 ± 9.82 **	−3.46 ± 7.69	−1.40 ± 9.01 **
L5 to pelvis	AP (mm)	−77.87 ± 8.18	−77.60 ± 8.05	−78.57 ± 7.70	−78.90 ± 7.15
	F/E(deg)	2.06 ± 12.93	3.22 ± 12.95 **	6.32 ± 8.52	7.20 ± 9.34 *

(AP, anterior/posterior axis; F/E, flexion/extension. * indicates a significant effect at *p* < 0.05, and ** indicates a significant effect at *p* < 0.01).

## Data Availability

The datasets generated and/or analyzed during the current study are not publicly available because the data are confidential patient data but are available from the corresponding author upon reasonable request.

## Supplementary Materials

The following supporting information can be downloaded at: https://www.mdpi.com/article/10.3390/bioengineering12030214/s1, Figure S1: Changes of L1 to the pelvis 6DOF kinematics before and after fatigue in patients with low back pain; Figure S2: Changes of L2 to the pelvis 6DOF kinematics before and after fatigue in patients with low back pain; Figure S3: Changes of L3 to the pelvis 6DOF kinematics before and after fatigue in patients with low back pain; Figure S4: Changes of L4 to the pelvis 6DOF kinematics before and after fatigue in patients with low back pain; Figure S5: Changes of L5 to the pelvis 6DOF kinematics before and after fatigue in patients with low back pain; Figure S6: Changes of L1 to the pelvis 6DOF kinematics before and after fatigue in healthy individuals; Figure S7: Changes of L2 to the pelvis 6DOF kinematics before and after fatigue in healthy individuals; Figure S8: Changes of L3 to the pelvis 6DOF kinematics before and after fatigue in healthy individuals; Figure S9: Changes of L4 to the pelvis 6DOF kinematics before and after fatigue in healthy individuals; Figure S10: Changes of L5 to the pelvis 6DOF kinematics before and after fatigue in healthy individuals.
