# Supplementary figures and images for: Different Spatial Characteristic Changes in Lumbopelvic Kinematics Before and After Fatigue: Comparison Between People with and Without Low Back Pain
Source: Bioengineering (Basel). 2025 Feb 20;12(3):214. doi: 10.3390/bioengineering12030214 (PMC11939769; doi:10.3390/bioengineering12030214)

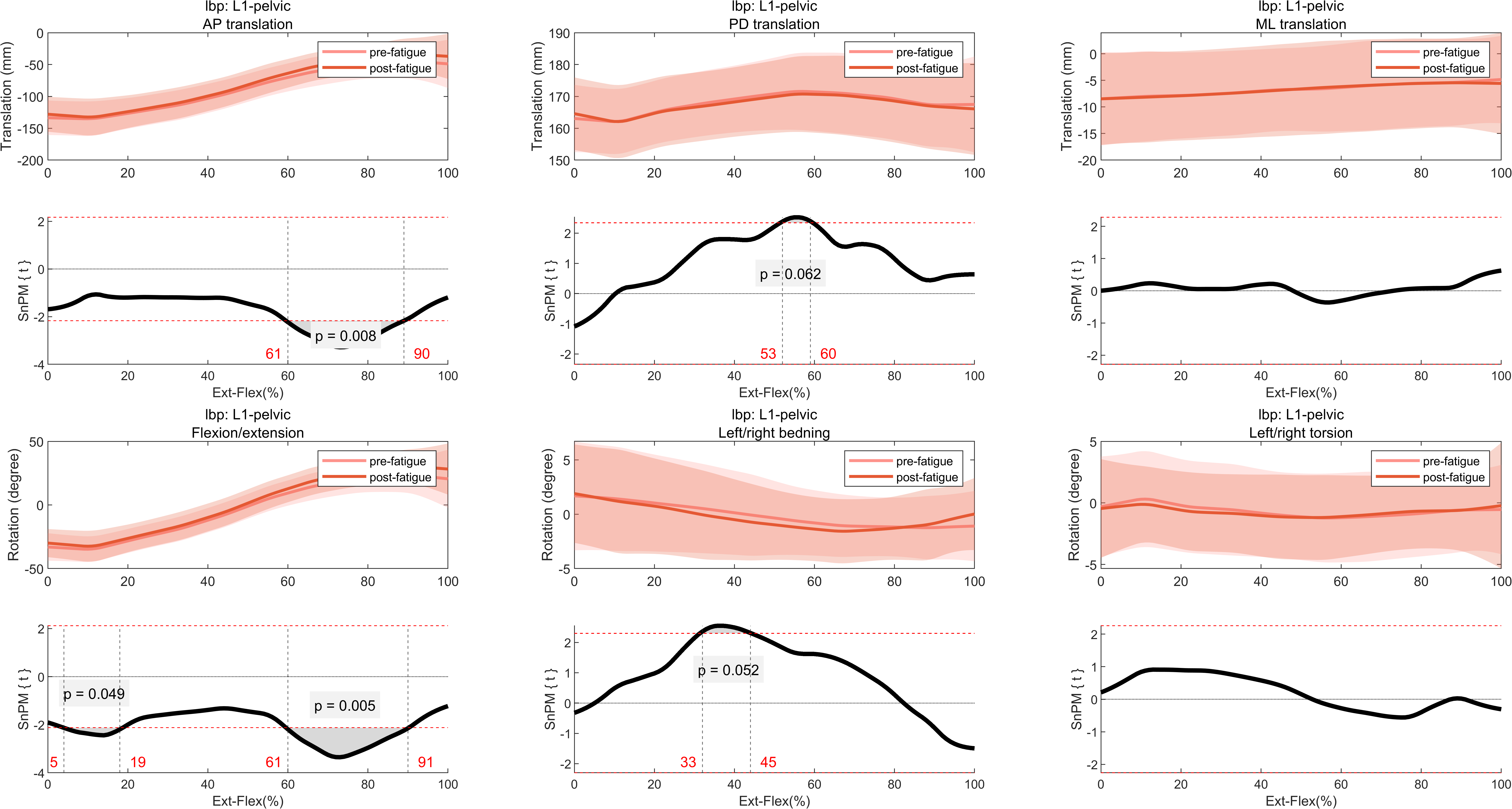

Supplement: Supplementary file 1 [file bioengineering-12-00214-s001.zip › Supplementary Files/Figure S1 lbp-L1-pelvic.png]

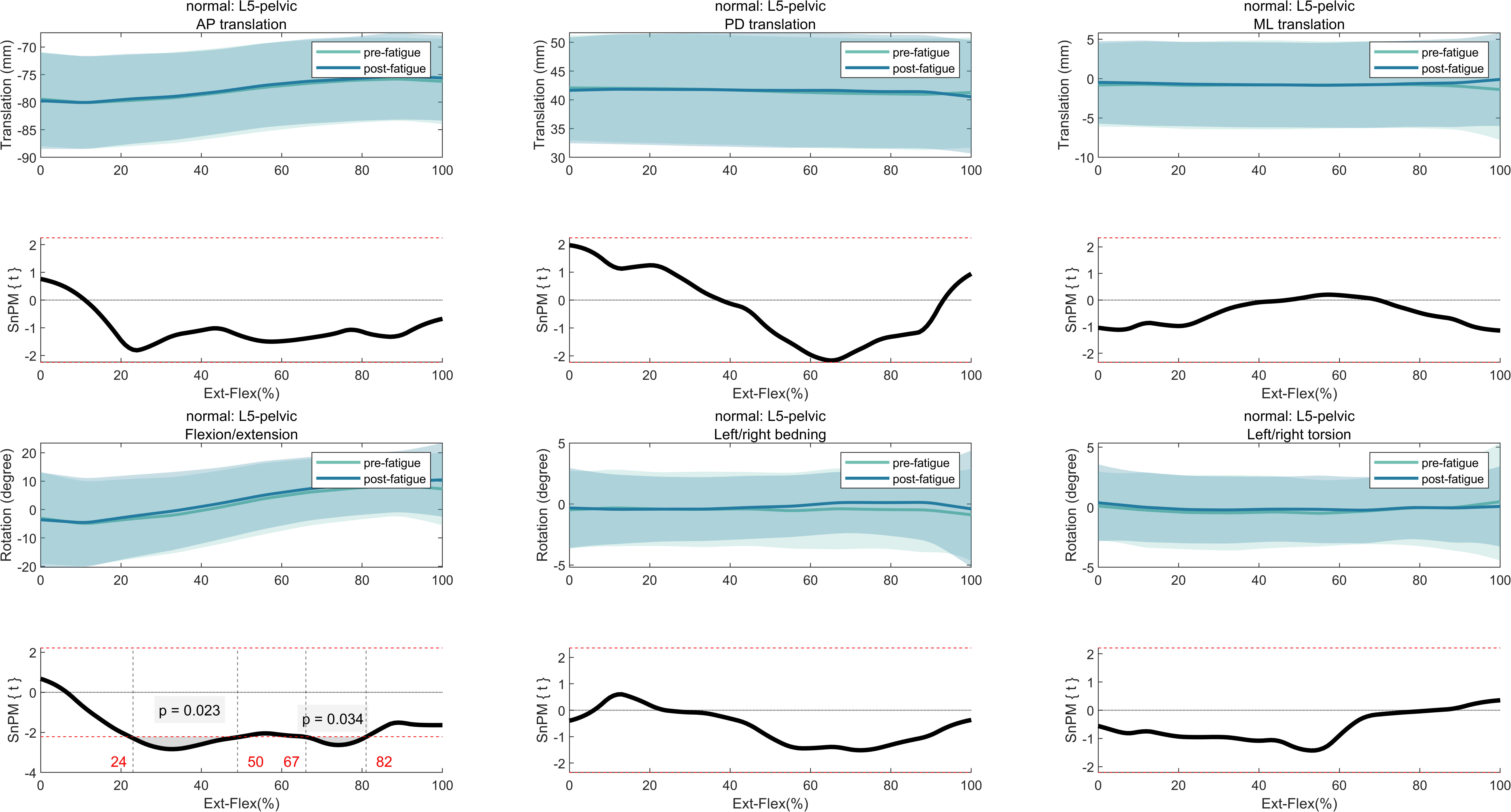

Supplement: Supplementary file 1 [file bioengineering-12-00214-s001.zip › Supplementary Files/Figure S10 normal-L5-pelvic.png]

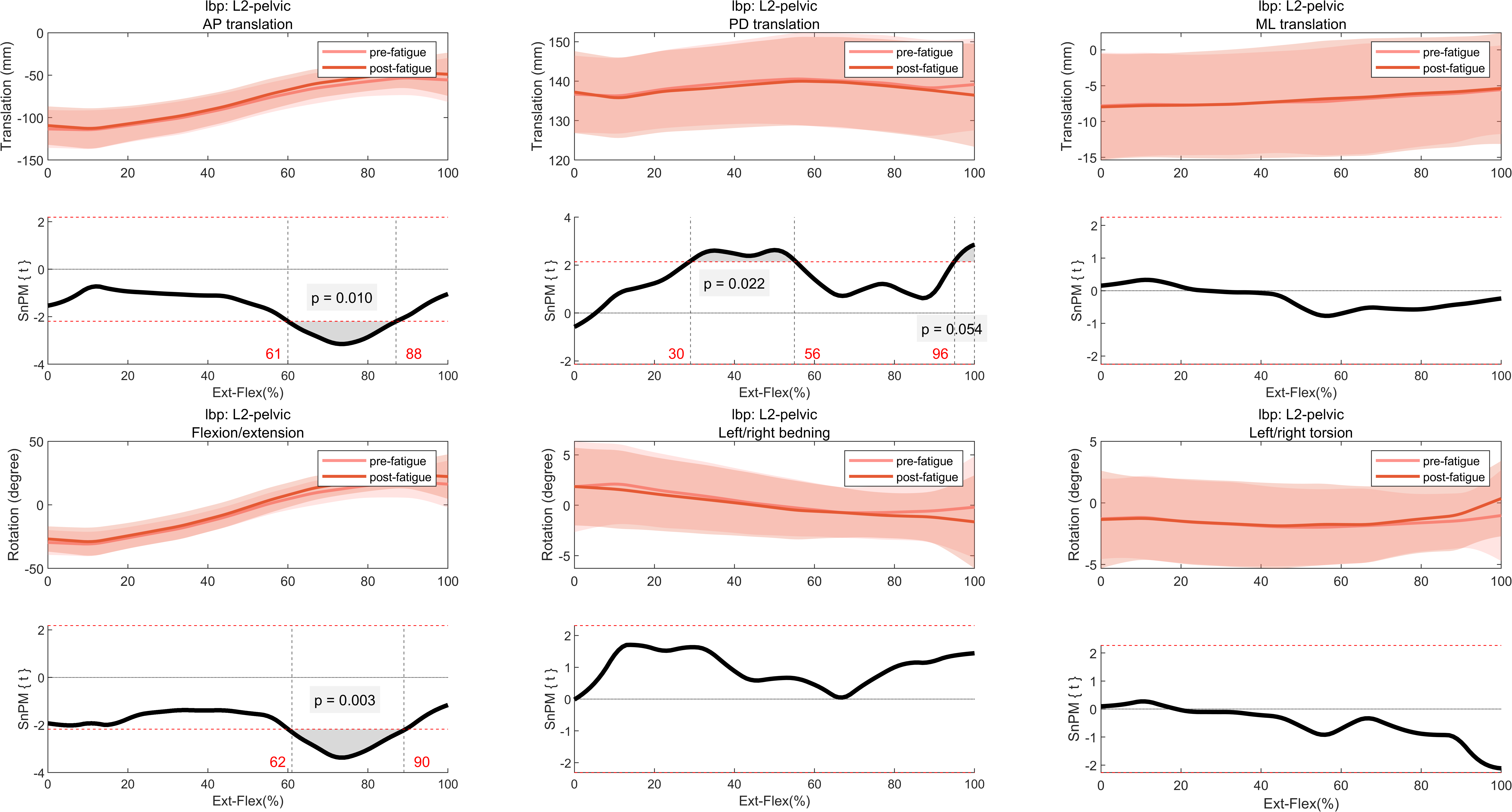

Supplement: Supplementary file 1 [file bioengineering-12-00214-s001.zip › Supplementary Files/Figure S2 lbp-L2-pelvic.png]

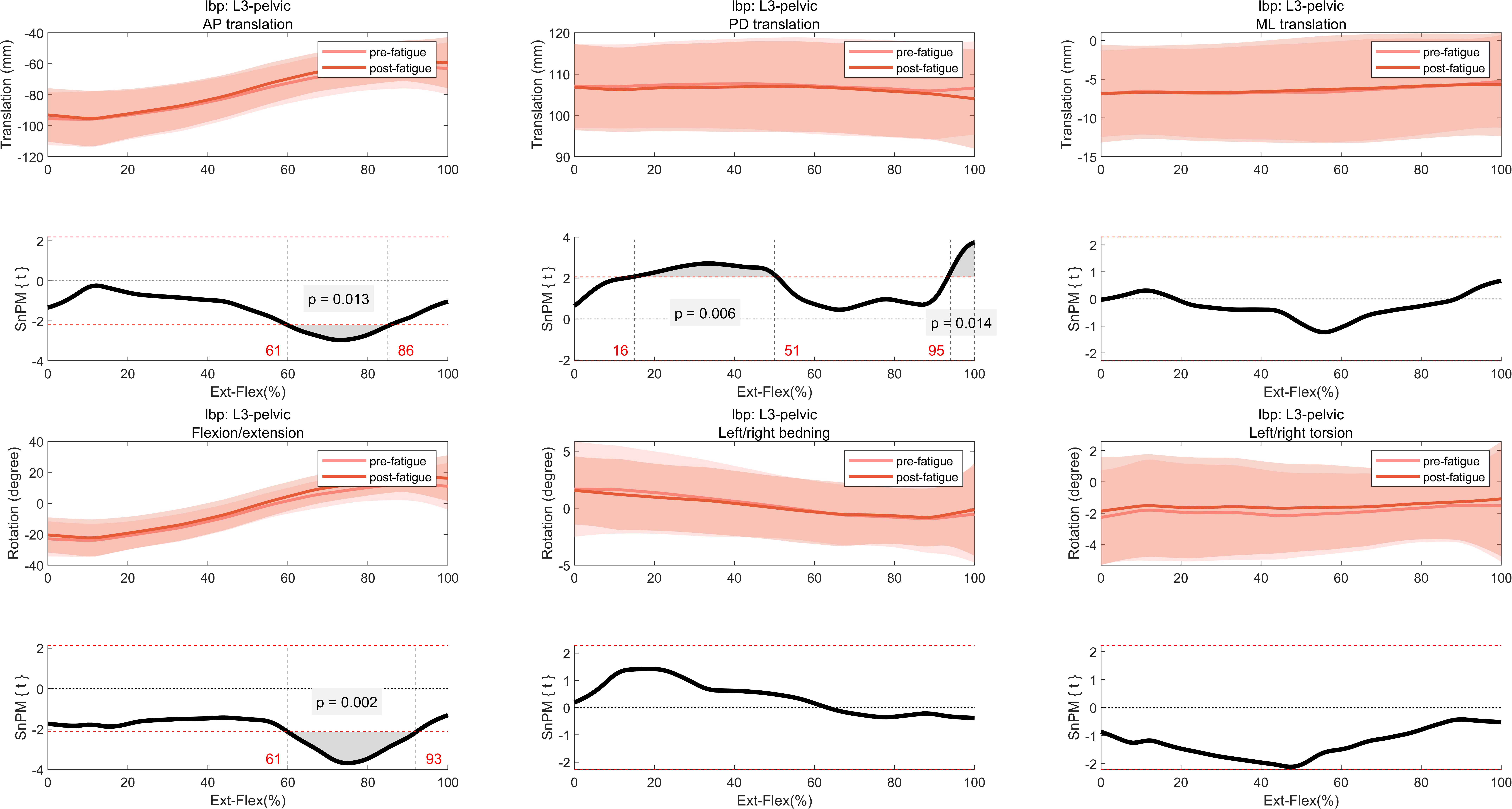

Supplement: Supplementary file 1 [file bioengineering-12-00214-s001.zip › Supplementary Files/Figure S3 lbp-L3-pelvic.png]

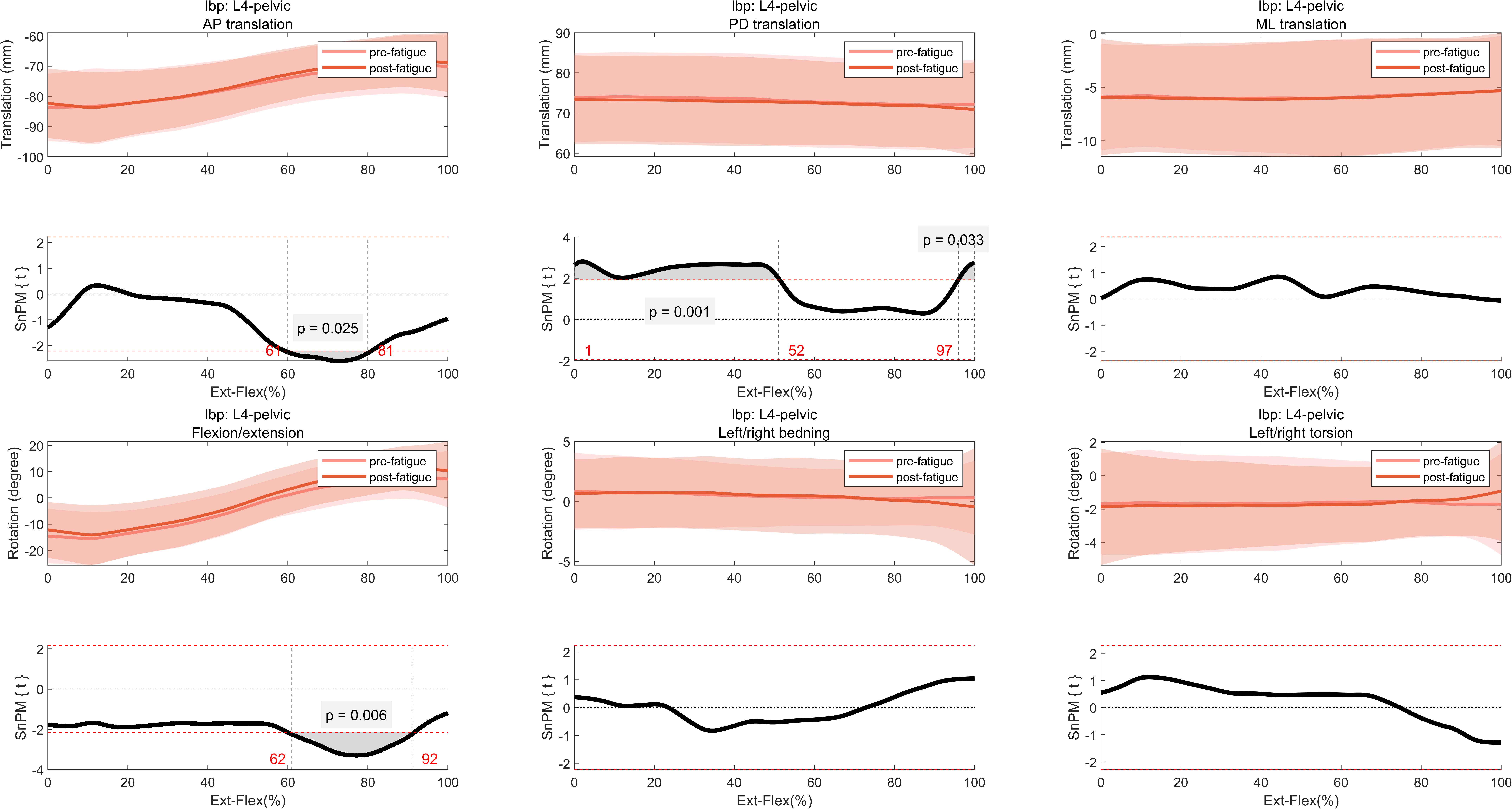

Supplement: Supplementary file 1 [file bioengineering-12-00214-s001.zip › Supplementary Files/Figure S4 lbp-L4-pelvic.png]

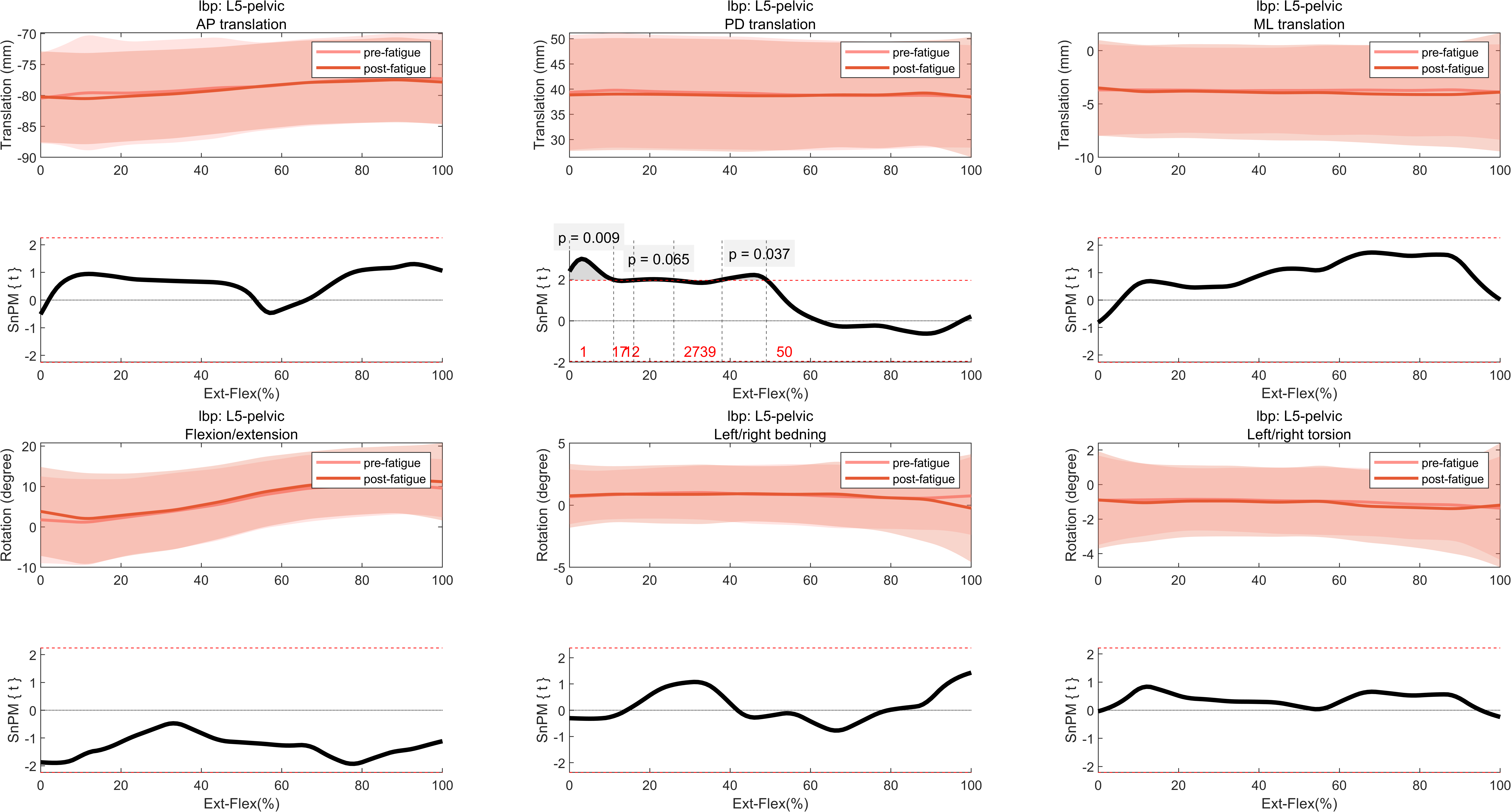

Supplement: Supplementary file 1 [file bioengineering-12-00214-s001.zip › Supplementary Files/Figure S5 lbp-L5-pelvic.png]

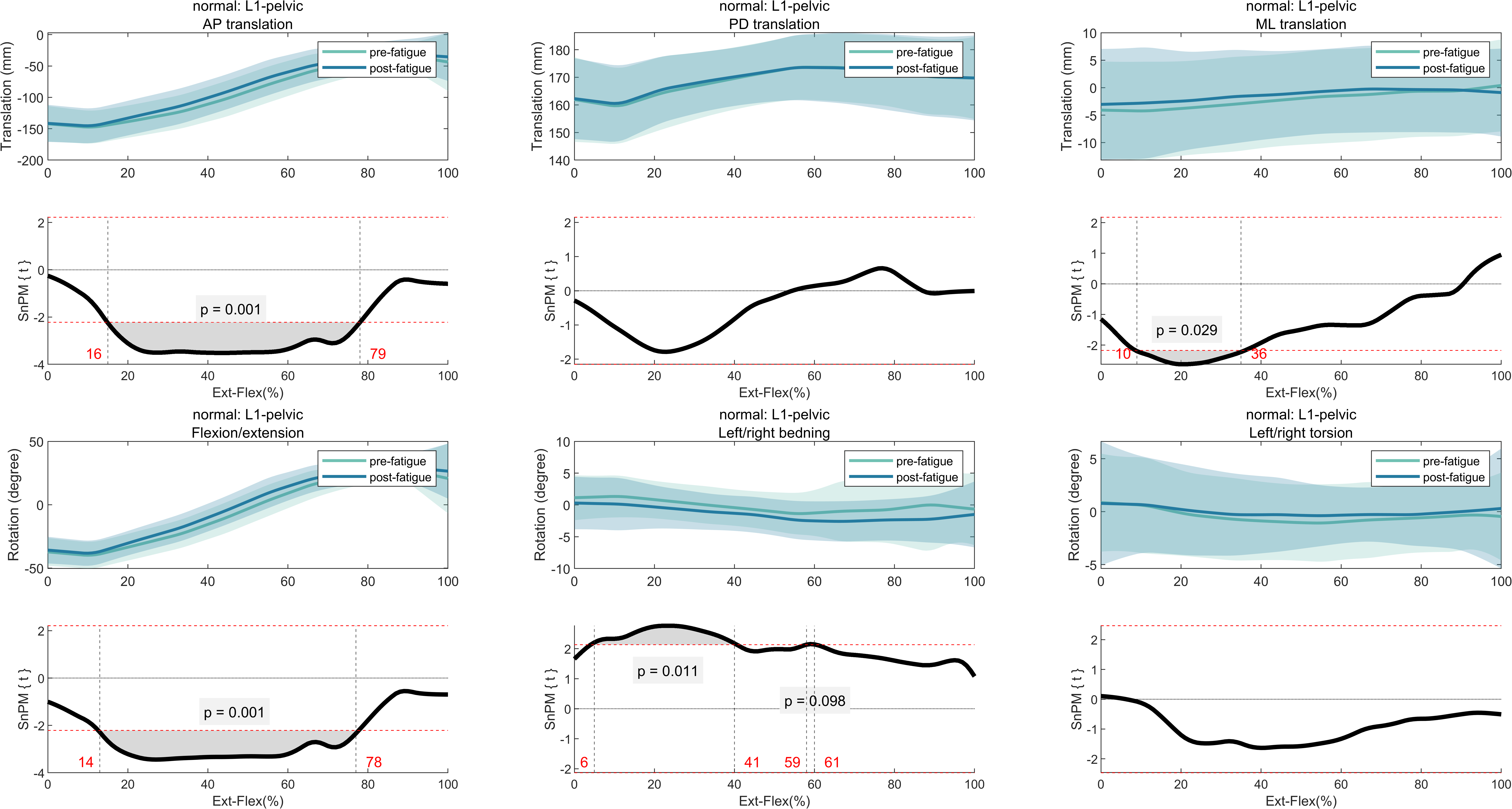

Supplement: Supplementary file 1 [file bioengineering-12-00214-s001.zip › Supplementary Files/Figure S6 normal-L1-pelvic.png]

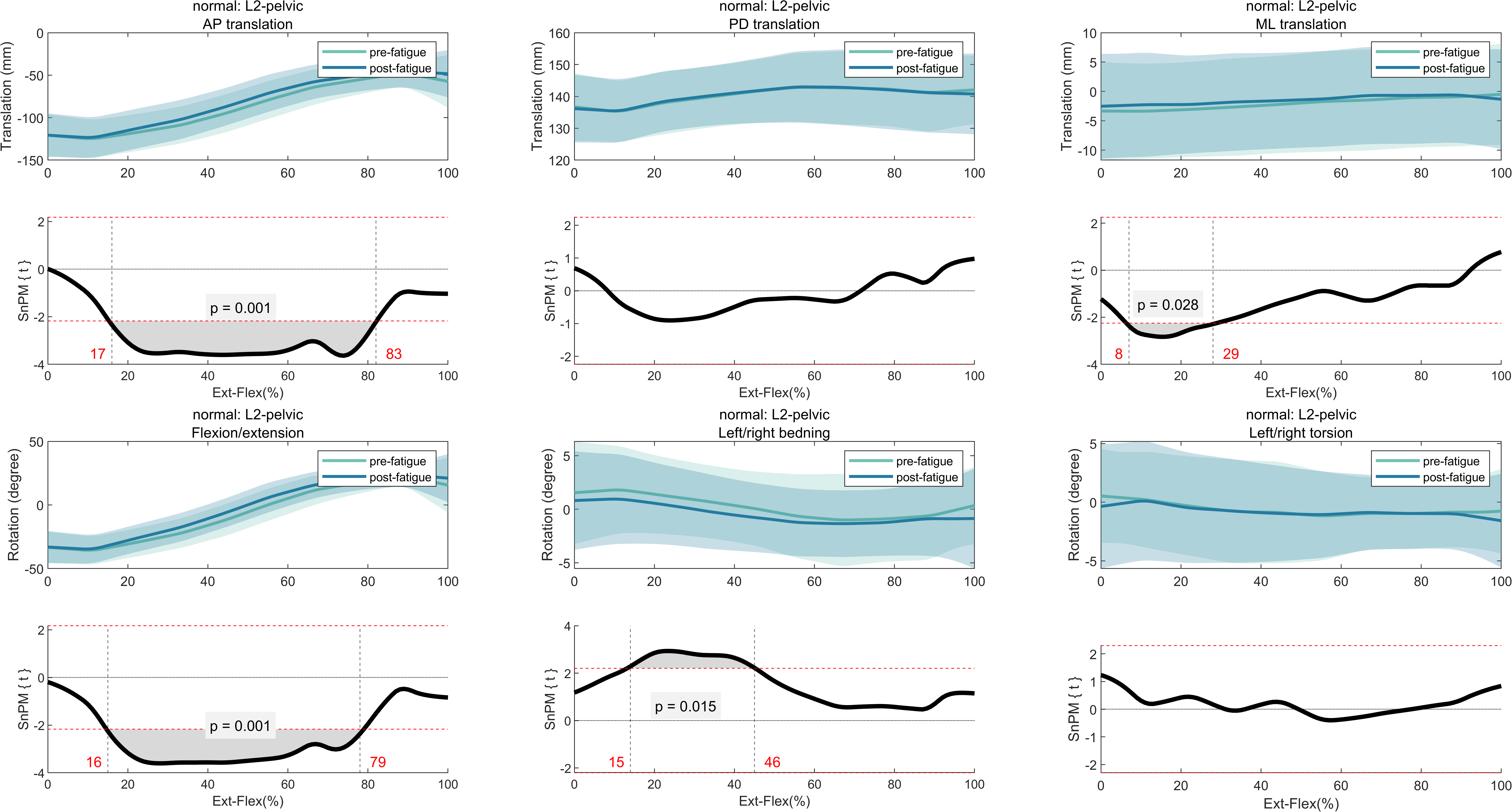

Supplement: Supplementary file 1 [file bioengineering-12-00214-s001.zip › Supplementary Files/Figure S7 normal-L2-pelvic.png]

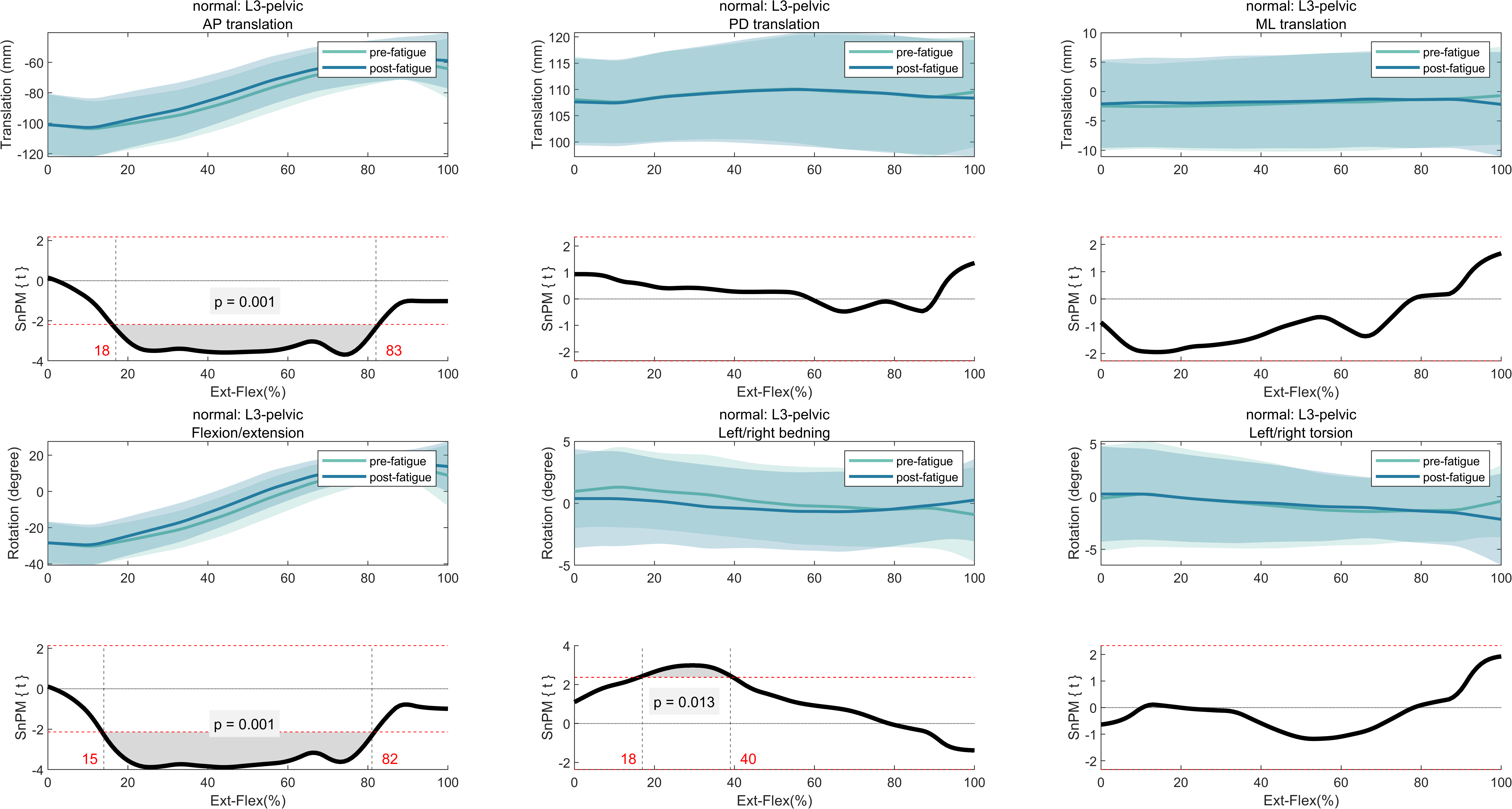

Supplement: Supplementary file 1 [file bioengineering-12-00214-s001.zip › Supplementary Files/Figure S8 normal-L3-pelvic.png]

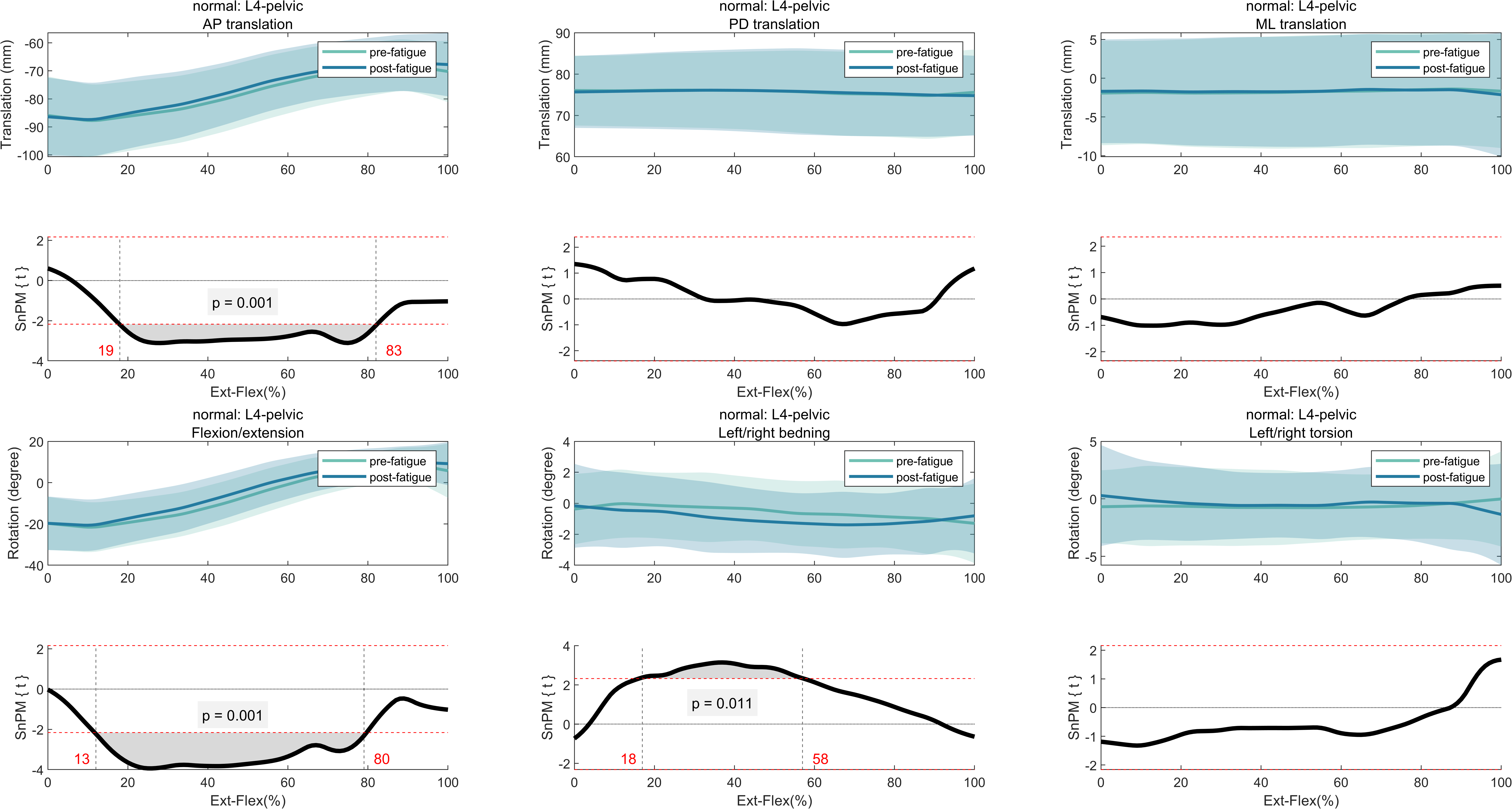

Supplement: Supplementary file 1 [file bioengineering-12-00214-s001.zip › Supplementary Files/Figure S9 normal-L4-pelvic.png]
